# Developing a scale for examining the perspective of university students on parental care

**DOI:** 10.3389/fpsyg.2023.1256110

**Published:** 2023-11-06

**Authors:** Xuxin Peng, Hisae Nakatani, Huifang Chen, Yuriko Inoue, Fang Song, Mikako Yoshihara, Ruxin Lei

**Affiliations:** ^1^Department of Community and Public Health Nursing, Graduate School of Biomedical and Health Sciences, Hiroshima University, Hiroshima, Japan; ^2^School of Nursing, Guangzhou Medical University, Guangzhou, Guangdong, China

**Keywords:** parental care, scale development, Japan, university students, public health

## Abstract

**Introduction:**

With the declining birth rate and increasingly aging population in Japan, an increased care burden may be placed on the family and the younger generation will address challenging circumstances when they can care for their parents. This study aimed to develop a scale for examining the perspectives of Japanese university students on parental care and determines its reliability and validity.

**Methods:**

A web-based survey on a total of 408 Japanese students was adopted. This study performed exploratory and confirmatory factor analyses to obtain the underlying factors of the scale. Reliability was verified using Cronbach’s α coefficient and Spearman–Brown’s split-half reliability α coefficient. Validity was verified through sample, criterion-related, and convergent and discriminant validity.

**Results:**

In total, the study identified a three-factor 11 item-scale. Cronbach’s α for the scale was 0.901, and the Cronbach’s α and split-half reliability α coefficients of each factor were greater than 0.7. Three factors explained 64.6% of the total variance. The model indicators were χ2/df = 2.241, comparative fit index (CFI) = 0.951, incremental fit index (IFI) = 0.951, TLI = 0.942, root mean square error of approximation (RMSEA) = 0.078 (*p* < 0.001). Thus, the study confirmed that the convergent and discriminant validity is acceptable. Correlations were noted for criterion-related validity (*r* = 0.675, *p* < 0.001).

**Discussion:**

The results show that the scale for examining the perspective of Japanese university students on parental care was confirmed with good reliability and validity.

## 1. Introduction

With Japan’s declining birth rate and increasingly aging population, the aging rate of people over 65 years was the highest worldwide (28.4%) and is expected to increase to 38.3% in 2055 (UN Population Division, 2019). Older people are more likely to suffer from multiple chronic diseases and disabilities, which may render them more likely to require assistance in their daily lives (Mitsutake et al., 2019).

In Japan, providing care for older adults is customary among children, that is, they bear the duty and responsibility to support their parents, which strongly emphasizes the parent–child bond (Nishioka, 2000). Caring for parents when they cannot do so for themselves is one of the most important principles derived from filial piety (Lum et al., 2016), which impacts children. Thus, they are more likely to care for their parents. However, due to new social trends, major demographic changes, a significantly declining birth rate, and an increasingly aging population (Someya, 2016), the need for intervention in public services is increasing as children face difficulties in adopting the responsibility of parental care (Muramatsu and Akiyama, 2011). Japan’s long-term care insurance, which was established in 2000, under the tagline of “from family care to societal care,” which emphasizes the participation of society in aging care (Tamiya et al., 2011). Compared with other East Asian societies that adhere to filial piety, a deep cultural division from the traditional norms of filial piety exists in Japan (Takagi and Saito, 2013), where children could be left to provide care for their older parents alone, which could be detrimental to their wellbeing.

The total fertility rate in Japan has also decreased from 2.135 in 1970 to only 1.42 in 2018, which has continued to decline (The World Bank, n.d.). A demographic trend demonstrates progressively fewer family caregivers per older person, which may place an increased burden on the family. When the younger generation reaches the age when they can care for their parents, they must, therefore, address challenging circumstances. The contentious debate between traditional standards of parental care and the socialization of care, which influences public policy, has emerged as a major social issue in Japan as a result of the decreasing birth rate but increasing aging population of the country.

Although the younger generation is more likely to take care of their families in the future, previous studies have found that perspectives on parental care have changed rapidly over the past few decades, which has raised concerns about the future care for their parents (Warmenhoven et al., 2018). A few studies have focused on filial piety in Japan (Tsutsui et al., 2014; Aires et al., 2019); however, less research is conducted on the perception of the younger generation, who will be future caregivers of the older adults, on caring for their parents (Goldberg-Looney et al., 2017). Bifarin et al. (2022) found that there is an anticipated dilemma between balancing work commitments and providing care due to cultural duty. Responsibility for parental care may arouse willingness, but it results in the lack of alternatives and a high possibility for burnout (Quinn et al., 2010). Furthermore, caring for older adults can influence the social connection and well-being of family caregivers (Al-Janabi et al., 2018).

Previous studies have noted that university students grew up in a different era than their parents (Sung, 2004), they live in a more multicultural society and are receiving higher education, they were more likely to be liberated from such traditional norms of sacrificing their life and care for their aging parents regardless of their physical and mental condition (Tsai et al., 2008). As a result, research that focuses on adult university students is important, because they compose the next generation who will be responsible for supporting the older adults and are considered important for the promotion of a welfare society. The discrepancy in low birth rate, aging population, and parental care between traditional norms and public policy is a significant social problem. With the rapid promotion of the welfare system, focusing on how the welfare system can take charge of parental care in the future is important.

In this study, the definition of “parental care” refers to the children providing daily care for their older parents (Kikuzawa and Uemura, 2021), which is intended to capture the cultural context and highlight the direction of care and intergenerational bond. Referring to the characteristic of family care behavior, the definition of “care” in the current study refers to the necessary daily care, such as dealing with meals, cleaning, excretion, laundry, medication, and so on (Peng et al., 2021).

This study aims to develop a scale for examining the perspectives of Japanese university students on parental care as well as the reliability and validity of the care, as these students will be the main force of parental care in the future.

## 2. Methods

### 2.1. Study design

This study was conducted to develop and validate the scale for the perspectives of Japanese university students on parental care.

### 2.2. Item generation

To inductively generate the items of the instruments, we conducted a qualitative survey (Borloti et al., 2017). From December 2021 to July 2022, we conducted a semi-structured interview survey on 19 Japanese university students using focus group interviews. The following information was obtained (1) What do you think about taking care of your parents?, (2) What is important for you in caring for your parents?, and (3) What are your perceptions and opinions about the use (or non-use) of care services when caring for your parents.

All interviews were recorded and transcribed. A context-focused content analysis of the transcripts was performed following the approach described by Graneheim and Lundman (2004). Data were categorized and organized into fundamental units relevant to the objective of the study. As a result, we hypothesized that the perspectives of Japanese university students on caring for their parents would be based on these four concepts and would intercorrelate, and the four concepts were as follows: “Responsibility of care for parents as the role of children,” “Beliefs about parental care,” “Distrust in public services” and “Balance between parental care and one’s life.” Furthermore, to ensure the rigor of the analyses, the study employed participant checks and expert reviews. According to the qualitative survey and a previous study (Van der Pas et al., 2005; Lowenstein and Daatland, 2006; Jones et al., 2011), 19 items of the scale draft were extracted.

### 2.3. Content validity test

This study validated the 19-item scale through a panel of five experts who confirmed if the items measure the relevant concept, whether or not they should be revised, and provided comments on modifying the items. The experts were selected using the following inclusion criteria: (1) with a master’s degree or above, (2) with more than 10 years of work experience involving the areas of public health or family care, (3) with more than 5 years of teaching experience in university; (4) with experience on scale development and validation. Specifically, they focused on determining whether the items (1) were correctly expressed, (2) reflected perspectives of parental care, (3) should remain on the scale, and (4) presented any bias in the content. It also examined whether or not item content was related to one’s perspective of parental care.

The study employed the item content validity index (I-CVI) and scale content validity index (S-CVI) to evaluate content validity. S-CVI > 0.90, and I-CVI = 1.00 indicated good validity when there were five experts participated (Polit and Beck, 2006). After the first round of consensus, the study obtained an S-CVI value of 0.58, and eight items obtained I-CVI < 1. After expert opinions, three items were added to the scale, four items were omitted, and four items were revised according to the suggestions of the experts. The final version of the scale was arranged and consisted of 18 items. All items reached I-CVI and S-CVI of 1, which indicated total agreement among the experts.

### 2.4. Face validity test

Ten students from a university in Japan performed a face validity test. The participants filled out a questionnaire, which asked them whether or not the meaning of an item was clear and understandable and whether or not any ambiguity or difficulty exists in responding. The results demonstrated that no problems were experienced in understanding or responding to the 18 items.

### 2.5. Participants and sample size

The participants were adult university students who met the following inclusion criteria: (1) were adult university students, (2) were Japanese people, and (3) were currently enrolled in that university. The exclusion criteria were those who were in graduate school and who were foreign students. Students were from two universities, one of a public school and one of a private school, and both of them were in urban areas. Students were divided into two groups: one for exploratory factor analysis (EFA) and the other for confirmatory factor analysis (CFA). EFA requires at least 10 subjects per item (Carneiro, 2003), and CFA requires a minimum of 200 participants (Anderson and Gerbing, 1984). Therefore, the study needed to recruit a total of 380 students with a minimum of 180 for EFA and 200 for CFA. The participants were randomly assigned to the EFA group or the CFA group by using simple randomization. Randomization was computer-generated using SPSS Ver.27.

### 2.6. Data collection

The questionnaire, informed consent, and QR code for the online survey questionnaire were distributed to the students after class. The online survey questionnaire was made using Google Forms (a secure web-based survey platform provided by Google). The subjects were requested to complete all forms online. The data collection period lasted from April 2023 to May 2023.

### 2.7. Measures

The survey included basic information, items on perspectives of parental care, and one criterion tool.

#### 2.7.1. Basic information

The basic information included gender, school years, siblings, family finances, presence of older adults in the family, family caregiving experience, and nursing knowledge.

#### 2.7.2. Perspective of the university students of parental care

The participants rated each item of the questionnaire on their perspective on parental care using a five-point Likert scale (1 = *strongly disagree*, 5 = *strongly agree*).

#### 2.7.3. Criterion-related validity

The filial obligation scale was used as a measure of the co-existing criterion-related validity with the permission of the creators (Ohta and Kai, 2002). This scale measures the filial obligation of children, which consists of three factors, namely, support for economic stability, aid for emotional satisfaction, and physical support for health. The scale is composed 11 items, which were rated using a five-point Likert scale ranging from 5 = *I agree* to 1 = *I do not agree* with Cronbach’s α coefficient of 0.81 (Ohta and Kai, 2007).

### 2.8. Data analysis

IBM SPSS Statistics for Windows Ver.27 (IBM Corp., Armonk, NY, USA) and Amos Ver.27 (IBM Corp., Armonk, NY, USA) were used for analysis. The study compared the EFA and CFA of the participant characteristics using the chi-square test.

#### 2.8.1. Reliability

To examine the reliability, ceiling, and floor effects, the study examined inter-item and item–total (I–T) correlations. The ceiling and floor effects were set to mean + standard deviation above 5 points, and the floor effect was set to mean − standard deviation below 1 point (Yusoff et al., 2021). Highly correlated coefficients may influence the results, such that inter-item correlation was set to *r* > 0.75 (Yusoff et al., 2021). For the I–T correlations, *r* < 0.2 was set as the criterion for exclusion.

Furthermore, Cronbach’s α and the Spearman–Brown split-half reliability α coefficients for each factor and the overall scale were calculated, and the criterion for Cronbach’s α coefficient was set to >0.7 (McKelvie, 1998; Yusoff et al., 2021).

#### 2.8.2. Construct validity

To examine construct validity, the study once again employed EFA and CFA. The participants were randomly divided into two groups for EFA and CFA.

To confirm factor structure, the study performed EFA (maximum likelihood method and Promax rotation). Items with a factor loading of 0.4 or higher and whose Cronbach’s α coefficient did not significantly increase when omitted were adopted to confirm factor structure and to rationally and appropriately identify the factors (Cabrera-Nguyen, 2010).

CFA confirmed the validity of the scale factor structure and model. For the goodness-of-fit of the model, the study used the comparative fit index (CFI), the incremental fit index (IFI), the Tucker–Lewis Index (TLI), and the root mean square error of approximation (RMSEA). The criteria for the goodness-of-fit of the model were GFI > 0.9, AGFI >0.9, CFI > 0.9, RMSEA <0.08 (Hooper et al., 2008).

#### 2.8.3. Sample validity

The study confirmed sample validity using the Kaiser–Meyer–Olkin (KMO) index and Bartlett’s sphericity test. The suitable sample size for factor analysis can be confirmed when KMO > 0.5 and *p* < 0.05 through Bartlett’s sphericity test (Williams et al., 2010).

#### 2.8.4. Convergent and discriminant validity

To examine convergent validity, the study used combination reliability (CR) and average variance extracted (AVE). The scale is considered to possess good convergent validity when AVE > 0.5, CR > 0.7, and when 0.36 < AVE < 0.5, the scale is considered to possess acceptable convergent validity (Shrestha, 2021; Lu et al., 2023). Discriminant validity is compared using the square root of AVE and the correlation coefficient between factors. The discriminant validity was assessed by calculating the square root of the AVE for each construct. The rule of assessing discriminant validity is the square root of AVE value must be greater than the correlation between all other constructs (Sahoo, 2019).

#### 2.8.5. Criterion-related validity

To examine criterion-related validity, we examined the relationship between the developed and filial obligation scales using Spearman’s rank correlation coefficient. A value of *p* of 0.05 was used as the cut-off for the significance.

### 2.9. Ethical considerations

This study was approved by the Ethics Committee of the organizations with which the authors are affiliated. The written research explanation clearly detailed the objective of the research, method, freedom to participate or withdraw, and privacy protection. Informed consent was verified before filling out the questionnaire.

## 3. Results

### 3.1. Participant characteristics

The study collected a total of 408 questionnaires (valid response rate: 45.4%); Table 1 presents the characteristics of the participants. The average age of the participants was 20.3 ± 2.1 years. Regarding gender, the sample was composed of 110 men (27.0%), 293 women (71.8%), and 5 with gender unreported (1.2%). Regarding school years, the first, second, third, and fourth grades were composed of 131 (32.1%), 77 (18.9%), 125 (30.6%), and 75 (18.4%) students, respectively. Regarding siblings, 276 students have a brother or sister (67.6%), and 132 students have no sibling (32.4%). Regarding family finances, 58 students (14.2%) thought that their family had lots of spare money, 94 students (23.0%) thought that their family had some spare money, 184 students (45.1%) thought that their family had no spare money, 62 students (15.2%) thought that their family had some financial issue in daily life, and 10 students (2.5%) thought that their family was difficult in family finances. There are no significant statistical differences were found between the EFA and CFA participant characteristics by using the chi-square test.

**Table 1:** Participant characteristics.

Item		Total (*n* = 408)		EFA (*n* = 204)		CFA (*n* = 204)		*p*
		*n*	%	*n*	%	*n*	%	
Gender	Male	110	27.0	65	31.9	45	22.1	0.079
	Female	293	71.8	137	67.2	156	76.5	
	Unreported	5	1.2	2	1.0	3	1.5	
School year	First year	131	32.1	59	28.9	72	35.3	0.496
	Second year	77	18.9	40	19.6	37	18.1	
	Third year	125	30.6	68	33.3	57	27.9	
	Fourth year	75	18.4	37	18.1	38	18.6	
Siblings	Have	276	67.6	136	66.7	140	68.6	0.672
	Haven’t	132	32.4	68	33.3	64	31.4	
Family finances	Have lots of spare money	58	14.2	28	13.7	30	14.7	0.120
	Have some spare money	94	23	39	19.1	55	27.0	
	Have no spare money	184	45.1	92	45.1	92	45.1	
	Have some financial issue	62	15.2	39	19.1	23	11.3	
	Difficult in family finances	10	2.5	6	2.9	4	2.0	
Presence of the older adults in the family	Have	169	41.4	80	39.2	89	43.6	0.366
	Haven’t	239	58.6	124	60.8	115	56.4	
Family caregiving experience	Have	29	7.1	11	5.4	18	8.8	0.177
	Haven’t	379	92.9	193	94.6	186	91.2	
Nursing knowledge	Excellent	51	12.5	29	14.2	22	10.8	0.312
	Good	113	27.7	62	30.4	51	25.0	
	Average	101	24.8	43	21.1	58	28.4	
	Below average	111	27.2	56	27.5	55	27.0	
	Poor	32	7.8	14	6.9	18	8.8	

### 3.2. Item analysis

The study noted the ceiling effect for two items (Item 1: 4.24 ± 0.921; Item 5:4.00 ± 1.079), and no floor effect was found. The inter-item correlation coefficients that displayed values higher than 0.75 were between Items 13 and 14 (*r* = 0.806, *p* < 0.001) and between Items 17 and 18 (*r* = 0.773, *p* < 0.001). we omitted Items 14 and 18 following the opinions of the experts. Furthermore, we excluded one item according to the I–T correlation coefficient, which was <0.3 (Item 16: *r* = −0.069, *p* = 0.168).

### 3.3. Exploratory factor analysis

The study conducted EFA via maximum likelihood and Promax rotation. Prior to EFA, the study confirmed the validity of the sample using the KMO index and Bartlett’s sphericity test. The suitable sample size for analysis can be determined using a KMO index of >0.5 and Bartlett’s sphericity test of *p* < 0.05. In this study, the KMO value reached 0.869, and Bartlett’s sphericity test was *p* < 0.001, which indicate the good fit of the sample validity. Moreover, the Kaiser criterion was used to determine the optimal number of factors, the factors were extracted of which the eigenvalue was greater than 1 criterion (Kaiser, 1960). A three-factor structure that showed a value of ≥1.0 from the eigen analysis of the correlation matrix was adopted. After the repeated deletion of items with factor loadings of less than 0.4, we excluded two items, which led to the development of a three-factor scale that consists of 11 items (Table 2), which explained 64.6% of the total variance. As the final EFA, KMO reached 0.871, and Bartlett’s sphericity test was *p* < 0.001. Moreover, Cronbach’s α coefficients were 0.908, 0.804, and 0.717 (total α = 0.893).

**Table 2:** Exploratory factor analysis.

Item		Factor 1	Factor 2	Factor 3
Factor 1: Blood-based sense of mission on parental care (*α* = 0.908)				
6	I want to care for my parents myself because they gave birth to me.	0.939	−0.021	−0.075
2	I want to care for my parents myself in the role of a child.	0.893	0.113	−0.116
3	I want to do whatever I can to take care of my parents.	0.797	0.117	0.011
4	I want to care for my parents myself due to the blood bonds.	0.754	−0.114	−0.020
7	I want to care for my parents myself because I think it is right.	0.656	−0.021	0.239
Factor 2: Distrust of care by others (*α* = 0.840)				
8	I want to care for my parents myself because I do not trust the ability of caregivers.	−0.078	0.884	0.091
10	I want to care for my parents myself because I think the nursing home cannot care for my parent properly.	0.007	0.738	0.073
9	I want to care for my parents myself because of news about caregiver abuse and other problems.	0.184	0.676	−0.076
Factor 3: Impact of providing parental care (*α* = 0.717)				
11	I want to care for my parents myself even if it means giving up my personal time.	0.140	−0.041	0.802
15	I want to care for my parents myself even if it sacrifices myself.	0.297	−0.003	0.568
13	I want to care for my parents myself because I am concerned about what my relatives and neighbors think of me.	−0.291	0.240	0.529
Factorial correlation matrix		1.000		
		0.432	1.000	
		0.545	0.612	1.000

Factor extraction: maximum likelihood. Rotation: Promax rotation.

Factor 1 was conducted using five items and is named “blood-based sense of mission on parental care,” because it involved a sense of mission in terms of parental care due to the parent giving them life and a strong blood bond between parents and children. Factor 2 was conducted using three items and was called “distrust of care by others,” because it indicated the mistrust of other people on parental care, such as care workers and nursing homes. Factor 3 was conducted using three items and labeled “impact of providing parental care,” because it involved the impacts of children when caring for their parents, such as “giving up own life,” “sacrifice,” and “how others think of me.”

### 3.4. Reliability

The Cronbach’s α coefficient of the overall scale was 0.901, and each factor was 0.865, 0.856, and 0.810. The Spearman–Brown split-half reliability for the overall scale was 0.903, and each factor was 0.886, 0.867, and 0.774. All Cronbach’s α coefficients were > 0.7, which indicated good reliability.

### 3.5. Confirmatory factor analysis

**Figure 1:**
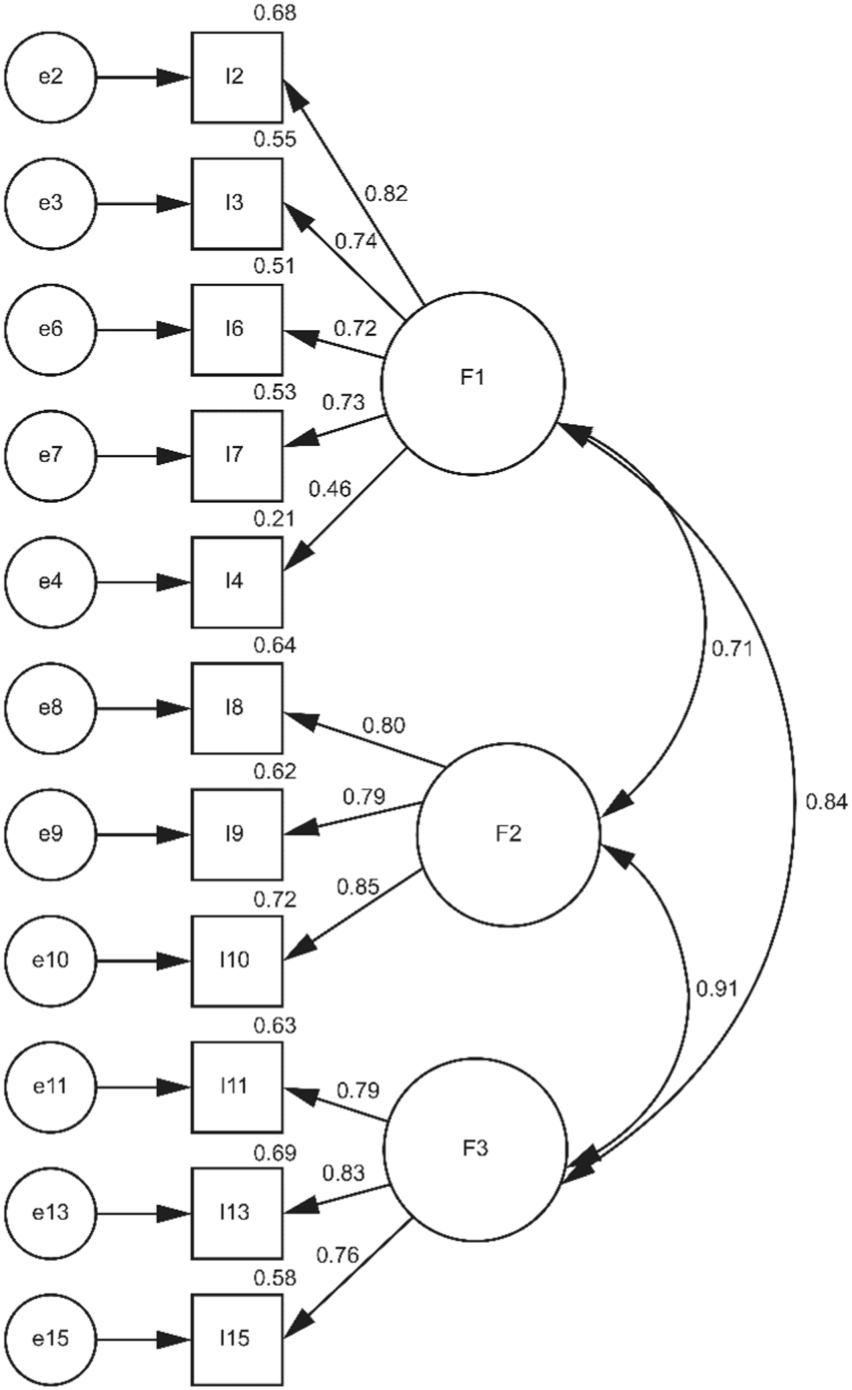
Confirmatory factor analysis. χ2/df = 2.241, CFI = 0.951, IFI = 0.951, TLI = 0.942, RMSEA = 0.078, *p* < 0.001. *F*, the number of each factor; I, the number of each item; e, error correlation for each item.

The study performed CFA to verify factor composition (11 items under three factors). The model demonstrated χ2/df = 2.241, CFI = 0.951, IFI = 0.951, TLI = 0.942, RMSEA = 0.078 (*p* < 0.001), which indicates a reasonable fitness (Figure 1).

### 3.6. Convergent and discriminant validity

For convergent validity, the AVE values for three factors were 0.493, 0.661, and 0.634, respectively; the CR for three factors were 0.825, 0.886, and 0.839. Moreover, the AVE values for two factors were > 0.5, and the other factor was in the range of 0.36 to 0.5. The CR of all factors were > 0.7, which indicate acceptable convergent validity (Table 3). For discriminant validity, the square root of AVE for each factor was greater than other correlation coefficients (Table 3).

**Table 3:** Convergent and discriminant validity.

	Factor 1	Factor 2	Factor 3
Factor 1	1.000		
Factor 2	0.460**	1.000	
Factor 3	0.538**	0.700**	1.000
√AVE	0.702	0.813	0.796
AVE	0.493	0.6608	0.634
CR	0.8252	0.886	0.839

**p < 0.01; Spearman’s rank correlation coefficient.

### 3.7. Criterion-related validity

For criterion-related validity, the study examined the correlation coefficient between the perspective on parental care and the filial obligation scales (Table 4). The study found a medium correlation between these two scales. The values were as follows: overall scale: *r* = 0.675 (*p* < 0.001), Factor 1: *r* = 0.643 (*p* < 0.001), Factor 2: *r* = 0.475 (*p* < 0.001), and Factor 3: *r* = 0.559 (*p* < 0.001). For the three factors of the perspective on parental care, there was a significantly positive correlation between Factor 1 and all the subscales of the filial obligation scales. And there was a significantly positive correlation between Factors 2 and 3 and the subscales of the filial obligation scales of “support for economic stability” and “physical support for health.” The values were as follows: (1) Factor 1: support for economic stability: *r* = 0.475 (*p* < 0.001), physical support for health: *r* = 0.598 (*p* < 0.001), aids for emotional satisfaction: 0.389 (*p* < 0.001); (2) Factor 2: support for economic stability: *r* = 0.396 (*p* < 0.001), physical support for health: *r* = 0.604 (*p* < 0.001); (3) Factor 3: support for economic stability: *r* = 0.493 (*p* < 0.001), physical support for health: *r* = 0.709 (*p* < 0.001).

**Table 4:** Criterion-related validity.

		Perspective on caring for their parents			
		Total	Factor 1	Factor 2	Factor 3
Filial obligation	Total	0.675**	0.643**	0.475**	0.559**
	Support for economic stability	0.545**	0.475**	0.396**	0.493**
	Physical support for health	0.756**	0.598**	0.604**	0.709**
	Aids for emotional satisfaction	0.237**	0.389**	0.070	0.083

**p < 0.01; Spearman’s rank correlation coefficient.

## 4. Discussion

This study aimed to develop a scale for evaluating the perspectives of Japanese university students on parental care. The results pointed to a three-factor structure of the 11 items. The three factors included “blood-based sense of mission on parental care,” “distrust in care by others,” and “impact of providing parental care.” Furthermore, the study tested for validity and reliability, which produced good results.

### 4.1. Contents of the scale

First, “blood-based sense of mission on parental care” included the sense of mission on parental care. In East Asia, loyalty to parents is influenced by Confucianism and traditional culture, which emphasizes dependence, obligation, and reciprocity in intergenerational relationships. Moreover, the obligation on parental care typically strengthens intergenerational bonds (Izuhara, 2010). In Japan, filial piety is defined as the tradition of respecting and caring for parents based on a moral obligation that children owe to their parents, and children were taught to care for their parents with dignity and respect (Hashimoto et al., 2005). Therefore, as a child, the missionary for parental care is an important part of measuring perspectives on parental care.

Second, “distrust of care by others” included mistrust in other people who are caring for their parents. Lin and Yi (2013) reported that adult children in East Asia, including Japan, were more likely to care for their parents by themselves, which is consistent with the findings of the current study. In Japan, family caregivers rarely use public services, because they believe in the family caregiving system, such that a sense of resistance and distrust exist toward the use of public care services and care resources outside the family (Tsukada and Saito, 2006). Additionally, whether care resources or caregivers outside the family are used plays an important role in the perspectives on parental care.

Third, “impact of providing parental care” indicated the impact such as repercussions, influence, and consequences on children when they care for their parents. In Japan, adult children are expected to take care of their parents and may face negative repercussions and sanctions from society and family if they do not adhere to this norm (Taniguchi and Kaufman, 2017). Moreover, social norms require children to sacrifice their time or others to support their parents as a repayment (Asai and Kameoka, 2005). These sacrifices may lead to adverse health outcomes, burden and stress, social isolation, and financial deprivation (Noguchi et al., 2020). Therefore, the impact of shouldering parental care is an important part of measuring perspectives on parental care.

### 4.2. Reliability and validity of the scale

The study examined the reliability and validity of the scale using EFA, CFA, and convergent, discriminant, and criterion-related validity. KMO reached 0.871, and Bartlett’s sphericity test was *p* < 0.001, which indicates good sample validity (Williams et al., 2010). EFA extracted the three-factor structure of the 11-item scale. Cronbach’s α coefficients were > 0.7 for the entire and all factors, which is the same as the split-half reliability coefficient, which indicated appropriate reliability (McKelvie, 1998; Yusoff et al., 2021). According to CFA, CFI, IFI, and TLI were > 0.9, and RMSEA was <0.08, which indicated an acceptable goodness-of-fit. In this study (Hooper et al., 2008), the AVE values were > 0.5 for two factors, greater than 0.36 but less than 0.5 for another factor, and all CR values for each factor were > 0.7, which indicate acceptable convergent validity (Shrestha, 2021; Lu et al., 2023). Furthermore, the square root of AVE for all factors was greater than all correlation coefficients, which indicated good discriminant validity (Sahoo, 2019).

In addition, the study verified criterion-related validity using the correlation between this scale and the filial obligation scale and predicted that a relationship exists between perspectives of parental care and filial obligation. As a result, the study found significantly positive correlations and inferred that filial obligation emphasized intergenerational bonds and guided children to follow the traditional norms of parental care. Moreover, Factor 1 showed significant correlations with the filial obligation scale and all the subscales of filial obligation. it’s inferred that the integrational blood-base bond strengthens filial obligations, which means children need to act to support their parents in most areas of their lives (Stuifbergen and Van Delden, 2011). Factors 2 and 3 showed significant correlations with the filial obligation scale and “Support for economic stability” and “Physical support for health,” which showed that the filial obligation emphasizes that children should care for their parents by themselves, even though the support and parental care may make an impact on their own life.

### 4.3. Implication of this study

The scale developed in this study helps family caregivers, health and social care practitioners, service commissioners, and service managers to ensure the perspective of Japanese university students on parental care and caregiving capacity within the family, which can guide them in developing a home care plan and improving healthcare services. Furthermore, as the highest-level-aging population in the world, the perspective on parental care among Japanese can provide implications and advice to other countries when they face a familiar situation like Japan in the future.

### 4.4. Limitations

This study has its limitations. First, only university students were included in the study; thus, extrapolating the results to all people in the younger generations is difficult. Second, other generations were beyond the scope of this study, but further research can focus on them to determine how other generations are exploring the differences between generations.

## 5. Conclusion

This study developed a scale for measuring the perspective of Japanese university students on parental care. Reliability and validity were confirmed, which comprises 11 items under three factors. The scale can be used to examine the perspective of parental care, improve caregiving capacity within families, and provide advice for home care service intervention.

## Data availability statement

The raw data supporting the conclusions of this article will be made available by the authors, without undue reservation.

## Ethics statement

The studies involving humans were approved by the ethics committee of epidemiology research at Hiroshima University (approval number E2022-0246). The studies were conducted in accordance with the local legislation and institutional requirements. The participants provided their written informed consent to participate in this study. Written informed consent was obtained from the individual(s) for the publication of any potentially identifiable images or data included in this article.

## Author contributions

XP: Conceptualization, Data curation, Formal analysis, Funding acquisition, Investigation, Methodology, Project administration, Resources, Software, Validation, Visualization, Writing – original draft, Writing – review & editing. HN: Conceptualization, Data curation, Formal analysis, Supervision, Validation, Writing – original draft, Writing – review & editing. HC: Supervision, Visualization, Writing – review & editing. YI: Data curation, Formal analysis, Validation, Writing – review & editing. FS: Data curation, Writing – original draft. MY: Data curation, Writing – original draft, Writing – review & editing. RL: Data curation, Writing – original draft, Writing – review & editing.
